# CHCHD2 up-regulation in Huntington disease mediates a compensatory protective response against oxidative stress

**DOI:** 10.1038/s41419-024-06523-x

**Published:** 2024-02-10

**Authors:** Xuanzhuo Liu, Fang Wang, Xinman Fan, Mingyi Chen, Xiaoxin Xu, Qiuhong Xu, Huili Zhu, Anding Xu, Mahmoud A. Pouladi, Xiaohong Xu

**Affiliations:** 1 Department of Neurology and Stroke Center, The First Affiliated Hospital of Jinan University, 613 Huangpu Avenue West, Guangzhou, Guangdong 510632 China; 2 Clinical Neuroscience Institute, Jinan University, 613 Huangpu Avenue West, Guangzhou, Guangdong 510632 China; 3 Department of Neurology, Taihe Hospital of Shiyan, Affiliated Hospital of Hubei Medical University, Shiyan, 442000 China; 4 Department of Plastic Surgery, The First Affiliated Hospital, Jinan University, 613 Huangpu Avenue West, Guangzhou, Guangdong 510632 China; 5 Department of Medical Genetics, Centre for Molecular Medicine and Therapeutics, Djavad Mowafaghian Centre for Brain Health, British Columbia Children’s Hospital Research Institute, University of British Columbia, Vancouver, V5Z 4H4 Canada

**Keywords:** Cell death in the nervous system, Neuroscience

## Abstract

Huntington disease (HD) is a neurodegenerative disease caused by the abnormal expansion of a polyglutamine tract resulting from a mutation in the *HTT* gene. Oxidative stress has been identified as a significant contributing factor to the development of HD and other neurodegenerative diseases, and targeting anti-oxidative stress has emerged as a potential therapeutic approach. CHCHD2 is a mitochondria-related protein involved in regulating cell migration, anti-oxidative stress, and anti-apoptosis. Although CHCHD2 is highly expressed in HD cells, its specific role in the pathogenesis of HD remains uncertain. We postulate that the up-regulation of CHCHD2 in HD models represents a compensatory protective response against mitochondrial dysfunction and oxidative stress associated with HD. To investigate this hypothesis, we employed HD mouse striatal cells and human induced pluripotent stem cells (hiPSCs) as models to examine the effects of CHCHD2 overexpression (CHCHD2-OE) or knockdown (CHCHD2-KD) on the HD phenotype. Our findings demonstrate that CHCHD2 is crucial for maintaining cell survival in both HD mouse striatal cells and hiPSCs-derived neurons. Our study demonstrates that CHCHD2 up-regulation in HD serves as a compensatory protective response against oxidative stress, suggesting a potential anti-oxidative strategy for the treatment of HD.

## Introduction

Huntington disease (HD) is a genetic neurodegenerative disease caused by the abnormal expansion of the CAG repeats encoding polyglutamine in the *HTT* gene resulting in the mutant huntingtin protein [1]. Currently, treatment options for HD are limited to symptom management, with no effective method to halt or delay disease progression. Research on HD pathogenesis holds great significance: the clear genetic basis of HD facilitates the establishment of animal and cellular models, providing a solid foundation for exploring underlying disease mechanisms and an opportunity to evaluate promising therapies in a well-defined patient population. Furthermore, HD shares clinical manifestations and molecular signaling pathway abnormalities with other neurodegenerative diseases like Alzheimer’s disease (AD) and Parkinson’s disease (PD), including specific subtypes of neuronal death and protein misfolding and deposition [2]. Therefore, investigating HD pathogenesis can offer important insights and ideas for the study and treatment of other neurodegenerative diseases.

The specific molecular mechanisms underlying HD pathogenesis remain incompletely understood, but previous studies have highlighted the involvement of oxidative stress in HD pathophysiology. For instance, increased oxidative stress has been observed in the peripheral blood of HD patients [3] and HD animal models [4]. Factors contributing to oxidative stress in HD include the aggregation of mutant huntingtin proteins, impaired antioxidant systems, elevated brain lipid content, high neuronal energy demands, mitochondrial electron transport chain damage, and mitochondrial dysfunction. HD cell mitochondria demonstrate significant alterations in morphology, structure, and Ca2^+^ homeostasis. These changes lead to reduced oxidative phosphorylation levels, inadequate ATP production, elevated levels of reactive oxygen species (ROS), and subsequent onset of oxidative stress. Excessive ROS levels are considered pathological markers of HD, inducing toxicity, contributing to further mitochondrial damage and protein misfolding, and ultimately resulting in neuronal death [5]. The nuclear factor NFE2-related factor 2-antioxidant response element (Nrf2-ARE) signaling pathway represents one of the most critical anti-oxidative stress pathways [6]. Activation of this pathway leads to the activation of various downstream antioxidant genes, including NQO1, GCLC, GCLM, and HO-1. These antioxidant genes eliminate cellular oxidative stress and restore the balance between pro-oxidants and antioxidants to maintain normal cellular function [7]. Importantly, Nrf2-ARE signaling activation has been identified in HD cells, potentially serving as a compensatory protective mechanism against oxidative stress in HD.

CHCHD2, a mitochondrial protein encoded by nuclear genes, belongs to the “CHCH” protein family. Its physiological functions are diverse, including regulation of cell migration, differentiation, oxidative phosphorylation, maintenance of mitochondrial structure, and anti-apoptosis [8]. Under hypoxic conditions, CHCHD2 acts as a transcription factor, binding to the conserved Oxygen Response Element (ORE) within the COX4I2 gene, and transcriptionally activating genes such as COX4I2 and CHCHD2 itself [9]. Knockout of the CHCHD2 gene in cells reduces COX activity, increases ROS levels, and leads to mitochondrial fragmentation and apoptosis [10, 11]. Conversely, overexpression of CHCHD2 protein reduces ROS levels and inhibits apoptosis [12], suggesting a potential role for CHCHD2 in anti-oxidative stress.

Although the physiological and pathological functions of the CHCHD family of proteins remain poorly understood, recent research has highlighted their close association with the development of neurodegenerative diseases [13]. Genetic analysis and experimental evidence indicate a strong correlation between *CHCHD2* gene mutations and Parkinson’s disease (PD) [14] and Lewy body disease (LBD) [15]. *CHCHD2* mutations in Drosophila have been shown to result in motor dysfunction, degeneration of dopaminergic neurons, shortened lifespan, and mitochondrial dysfunction [16]. Moreover, PD-associated *CHCHD2* mutations in human embryonic stem cells (hESCs) have been found to induce mitochondrial abnormalities, including hollow mitochondria with reduced cristae [17].

CHCHD2 has been shown to be up-regulated in HD patient peripheral blood cells, hESCs, human induced pluripotent stem cells (hiPSCs), and hESCs/hiPSCs-derived neuronal cells [18–22]. However, the precise role of CHCHD2 in HD, particularly in modulating HD phenotypes, remains unknown. Given its involvement in maintaining mitochondrial function and resisting oxidative stress, we hypothesize that the up-regulation of CHCHD2 expression may serve as a compensatory protective response to mitochondrial dysfunction and oxidative stress in HD. In addition, we propose that CHCHD2 exerts a neuroprotective effect through the Nrf2-ARE anti-oxidative stress signaling pathway. This study aims to test this hypothesis by manipulating CHCHD2 expression through overexpression or inhibition in HD mouse cell lines and hiPSC models. Moreover, we aim to elucidate the molecular mechanisms underlying the up-regulation of CHCHD2 expression and its anti-oxidative stress effects in HD. The findings from this study have the potential to contribute to the development of biomarkers and therapeutic drug targets for HD and other neurodegenerative diseases.

## Materials and methods

### Materials

All cell culture reagents were obtained from Gibco Life Technologies, while other reagents were sourced from Beyotime Biotechnology (China), unless otherwise specified. The GV112 lentiviral constructs containing mouse CHCHD2 shRNA (shCHCHD2) target sequence (5′-AAGTGTGGACCCTTATATT-3′) or a non-silencing control (shControl), as well as the GV348 lentiviral constructs containing mouse CHCHD2 (NM_024166) gene (CHCHD2-OE) or negative control (Vector), were designed, constructed, and packaged by Shanghai Genechem Co., Ltd (China). The shRNA against human CHCHD2 was obtained from TRC Lentiviral shRNA Libraries (TRC number: TRCN0000141561), with the sequence “5′-CCGGCAGTGGAGGAAGTAATGCTGACTCGAGTCAGCATTACTTCCTCCACTGTTTTTTG-3′“. The SHC016 Sigma MISSION® pLKO.1-puro non-Target shRNA plasmid was used as a scramble shRNA for human shCHCHD2. The resulting lentiviral constructs and packaging plasmids (pMDL, VSV-G, and pREV) were transfected into HEK293T cells using Lipofectamine 2000 (Life Technologies, USA). Lentiviruses were collected 48 h post-transfection and stored at −80 °C.

### Cell line culture

Immortalized striatal cell lines STHdh^Q7/Q7^ (wild type, Q7) cells, and STHdh^Q111/Q111^ (mutant, Q111) cells, expressing 7 and 111 glutamine repeats, respectively, were derived from HdhQ7/Q7 and HdhQ111/Q111 knock-in mice as described by [23]. Neuro-2a, SH-SY5Y, and HEK-293T cell lines were obtained from the American Type Center Collection (ATCC, USA). All cells were cultured in DMEM media (high glucose) supplemented with 10% fetal bovine serum, 100 U/ml penicillin, and 100 μg/ml streptomycin in 37 °C incubator with 5% CO_2_.

### Construction of stable cell lines

To construct stable strains, Q7 and Q111 cells were seeded in 24-well plates and infected with Vector, CHCHD2-OE, shControl, shCHCHD2 lentiviruses (Shanghai Genechem Co., Ltd, China) at a multiplicity of infection (MOI) of 20, respectively. After 48 h of infection, 1 μg/ml puromycin was added to the cultures to eliminate uninfected cells until all cells in the negative control group died. Subsequently, puromycin-resistant cells were expanded, and finally verified by Western blotting and quantitative real-time polymerase chain reaction (qRT-PCR).

### hiPSCs culture and neuronal differentiation

CAG180 HD hiPSCs, derived from dermal fibroblasts of a juvenile HD patient, were obtained from the NINDS Repository. The isogenic control cells (HD-C#1) were generated using the Clustered Regularly Interspaced Short Palindromic Repeat (CRISPR)-Cas9 genome editing method in our previous study [20]. hiPSCs were cultured with mTeSR™1 medium (STEMCELL Technologies) in Matrigel (Coring) coated plates. hiPSCs were differentiated into forebrain neurons using our previously published protocol [20, 22]. Briefly, hiPSCs were induced into neural progenitor cells (NPCs) in N2B27 medium (DMEM-F12/Neural Basal medium 1:1) supplemented with 1% N2, 2% B27, 1% non-essential amino acids, and 2 mM L-glutamine. This medium was further supplemented with specific small molecules and growth factors for a period of 15 days. The NPCs were then differentiated into forebrain neurons in N2B27 medium supplemented with 20 ng/ml BDNF, 20 ng/ml GDNF, 0.5 mM dbcAMP, and 0.2 mM ascorbic acid. The medium was half changed every 3–4 days during the terminal differentiation of neurons.

### Cell viability measurement

The cells were treated with tert-Butyl Hydroperoxide (TBHP, Macklin, China) or control liquid for 24 h and the cell viability were determined by CellTiter-Lumi™ Luminescent Cell Viability Assay Kit (Promega) according to the manufacturer’s instructions. The luminescence was measured using a Varioskan LUX multimode microplate reader (Thermo Fisher Scientific).

### RNA isolation and qRT-PCR

The total RNA was extracted with an RNA-Quick Purification Kit (ESscience, China), and 500 ng of total RNA was reverse transcribed into cDNA by PrimeScript™ RT Master Mix Kit (TaKaRa, RR036A). qRT-PCR was performed on CFX96 Touch Real-Time PCR Detection System (Bio-Rad) using TB Green® Premix Ex Taq™ kit (TaKaRa). The specific primer sequences used are shown in Supplemental Table 1. Relative gene expression levels were analyzed using the Comparative CT Method (ΔΔCt method).

### Western blotting

The cells were lysed using RIPA lysis solution, and protein quantification was performed using the Pierce™ BCA Protein Assay Kit (Thermo Fisher Scientific) for accurate measurement of protein levels. Lysates containing equal amounts of protein were loaded into each lane and separated on 6% (for HTT protein) or 12% SDS-PAGE gels. Subsequently, they were transferred to a nitrocellulose membrane (GE Healthcare). Blotting of the membranes was carried out using primary antibodies, including anti-β-actin (3700S, Cell Sailing Technology, 1:1000), anti-CHCHD2 (19424-1-AP, Proteintech, 1:200), anti-HTT (MAB2166, Millipore, 1:100), anti-Calnexin (AF5362, Affinity Bioscience, 1:200), and anti-Cleaved Caspase-3 (AC033, Beyotime, 1:100). The membranes were then probed with Peroxidase-Conjugated Goat Anti-Rabbit/Mouse IgG (H + L) secondary antibodies (Yeasen Biotechnology, China). The protein bands were visualized using the Tanon 250 Gel Imaging System (TANON Science& Technology Co, China) and quantified using ImageJ software.

### Detection of mitochondrial and cellular ROS

The cells were detached with 0.25% Trypsin-EDTA, followed by tincubation with fluorescent dyes as instructed by the manufacturer. MitoSOX™ Red Mitochondrial Superoxide Indicator (MitoSox Red, Wuhan Yeasen Biotechnology, China) and 2′,7′-Dichlorodihydrofluorescein diacetate (H2DCFDA, MedChemExpress LLC, United States) were used to detect mitochondrial and cellular reactive oxygen species (ROS), respectively. The fluorescence emitted by the cells was measured using the BD FACSCanto™ II Flow Cytometer (BD Biosciences), and the data were analyzed using Flow Jo™ v10 software.

### Immunofluorescence staining

hiPSCs, hiPSC-derived NPCs and neurons cultured on coverslips were fixed with 4% paraformaldehyde (PFA) for 15 min. Subsequently, the cells were permeabilized using 0.3% Triton X-100 and blocked with a solution containing 3% normal donkey serum and 0.1% Triton X-100 in PBS. The cells were then incubated with primary antibodies, including anti-OCT4 (sc-5279, Santa Cruz, 1:500), anti-NESTIN (MAB5326, Millipore, 1:200), anti-PAX6 (PRB-78P, Convance, 1:300), anti-MAP2 (MAP3418, Millipore, 1:200), anti-DARPP-32 (sc-11365, Santa Cruz, 1:100), and anti-SYP (ab68851, Abcam, 1:100), overnight at 4 °C. Following this, the appropriate Alexa Fluor secondary antibodies were applied for 1 h at room temperature, followed by staining with 1 mg/mL DAPI (MBD0015, Sigma-Aldrich, USA) for 10 min. The images were acquired using an Olympus FV100 inverted confocal microscope.

### TUNEL assay

The hiPSC-derived neurons at day 40 were switched to N2B27 medium without growth factors (GF withdrawal) for 48 h. Subsequently, the cells were fixed with 4% paraformaldehyde (PFA) and subjected to TUNEL (terminal deoxynucleotidyl transferase-mediated dUTP-biotin nick end labeling) staining using the in-situ cell death detection kit (Roche) following the manufacturer’s instructions. For quantification, images were acquired from eight non-overlapping fields on each coverslip using an Olympus FV100 inverted confocal microscope. Cell numbers were counted in a double-blinded manner, and the data used for analysis were obtained from three independent experiments.

### ARE-luciferase reporter assay

SH-SY5Y cells or Q7 cells were transfected with plasmids containing an ARE-dependent Firefly luciferase reporter gene and the Rinella luciferase gene, which served as a control for transfection efficiency. After 4 h of incubation, the cell medium was changed, and then the medium containing 50 μM TBHP was added and incubated for 20 h. The luciferase activity was measured using the Dual-Luciferase Reporter Assay System (Promega, E191) according to the manufacturer’s protocol.

### Statistical analysis

All statistical analyses were performed in GraphPad Prism 8 software. Two-way ANOVA analysis was used to test for differences across different groups, followed by Bonferroni post hoc multiple-comparison test for specific group comparisons. Otherwise, for the comparisons of the means between two groups, Student’s t-test was were applied. All data were presented as mean ± SEM and *p*-values were considered as follows: ns, no significance; *p < 0.05, **p < 0.01, ***p < 0.001, and ****p < 0.0001 for shown comparisons, and ^#^*p* < 0.05, ^##^*p* < 0.01, ^###^*p* < 0.001, and ^####^*p* < 0.0001 for comparisons relative to corresponding Q7 group.

## Results

### CHCHD2 up-regulation in HD cells

**Fig. 1:**
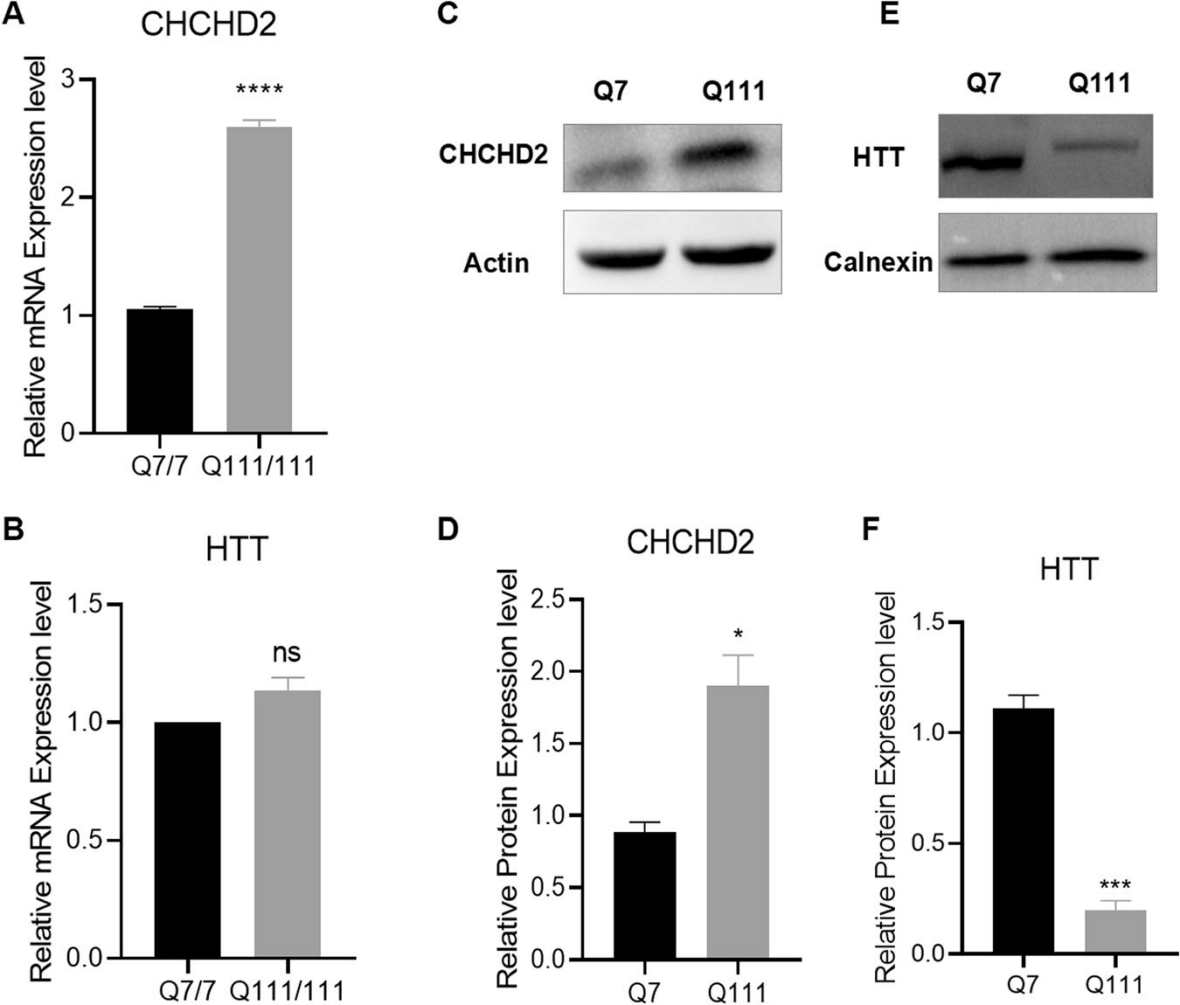
CHCHD2 is up-regulated in HD cells. mRNA expression levels of CHCHD2 (**A**) and HTT (**B**) were measured in Q7 (WT) and Q111 (HD) cells by qRT-PCR. **C** Representative western blot image for CHCHD2 protein expression in Q7 and Q111 cells, and the densitometric analysis was presented in (**D**). **E** Representative western blot image for total HTT protein expression in Q7 and Q111 cells, and the quantification of total HTT was shown in (**F**). *n* = 3 independent biological replicates. Values shown as mean ± SEM, and ns, no significance, *p < 0.05, ***p < 0.001, and ****p < 0.0001 was determined by unpaired student t-test.

hiPSCs derived from adult cells of HD patients contain endogenously expressed full-length mutated HTT proteins, making them an ideal model with the same genetic background as the patients. Our previous studies have demonstrated the up-regulation of CHCHD2 in HD iPSCs, and their derived NPCs [20, 22, 24] and neurons (Supplemental Fig. 1). However, the role of increased expression of CHCHD2 in HD pathogenesis remains unclear. To further investigate the effects of CHCHD2 on HD and its underlying mechanism, we utilized the murine immortalized striatal HD cell line Q111 and the corresponding wild-type (WT) line Q7 in this study. These cell lines have been widely used and offer ease of manipulation for experimental purposes. Initially, we validated the mRNA and protein expression levels of HTT and CHCHD2 in Q7 (WT) and Q111 (HD) cells (Fig. 1A, B). The results demonstrated a significant up-regulation of CHCHD2 in Q111 cells at both the mRNA and protein levels (Fig. 1A, C, D). Although the total HTT mRNA expression appeared similar between the WT and HD cells (Fig. 1B), the total HTT protein levels were decreased in HD cells compared to WT cells (Fig. 1E, F) as shown previously [25]. This finding is consistent with previous reports that CHCHD2 is up-regulated in human embryonic stem cells (hESCs), hiPSCs, and their derived neuronal cells [19, 22].

### CHCHD2 induction by oxidative stress in mouse and human neuronal cells

**Fig. 2:**
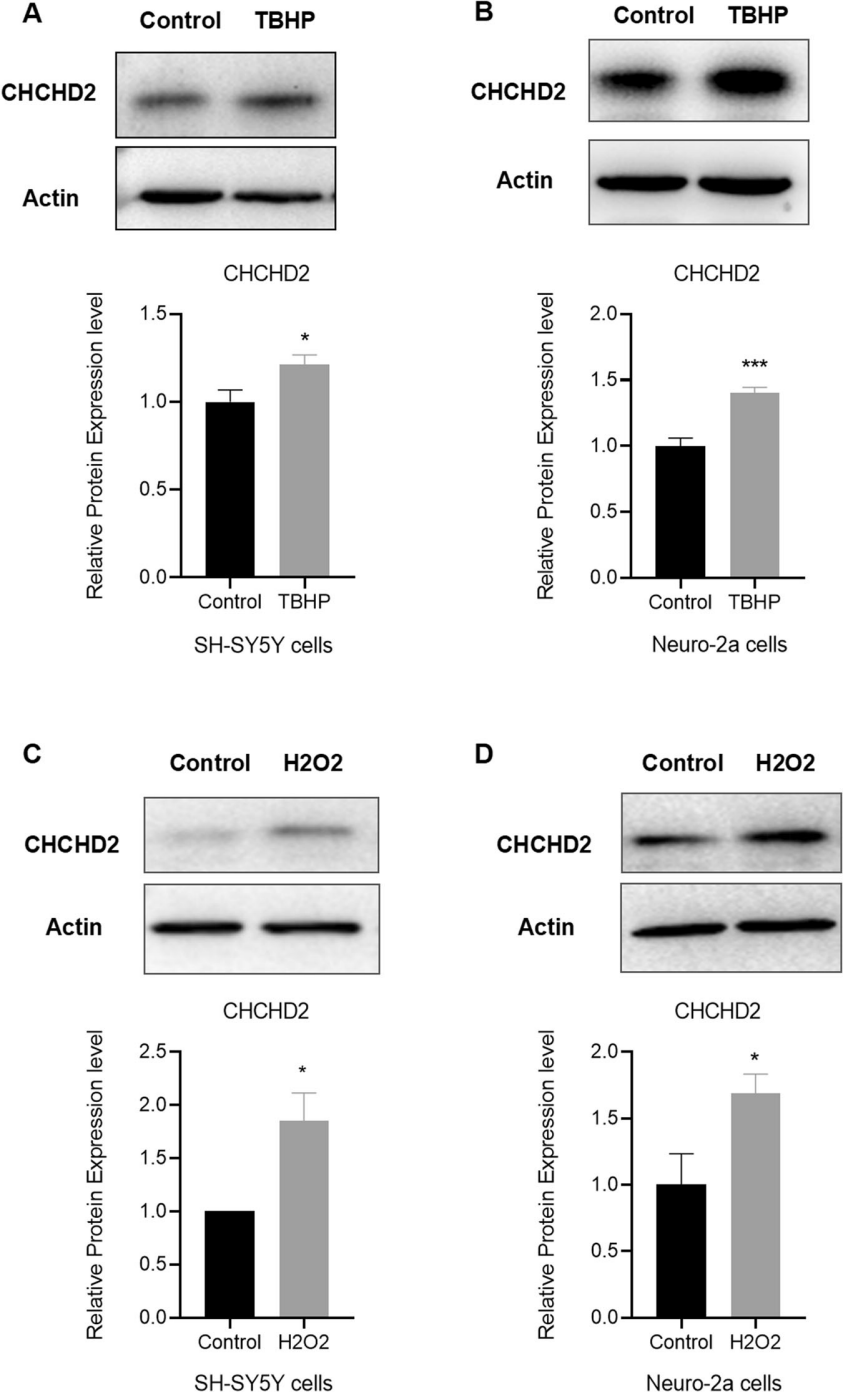
CHCHD2 is highly induced by oxidative stress in neuronal cells. **A**, **B** CHCHD2 expression is up-regulated after 24 h TBHP (100 µM) treatment in human SH-SY5Y cells (**A**) and mouse Neuro-2a cells (**B**). *n* = 6 biological replicates. **C**, **D** CHCHD2 expression is induced after 2 h H_2_O_2_ (25 µM) treatment in SH-SY5Y cells (**C**) and Neuro-2a cells (**D**). *n* = 4 biological replicates. Values shown as mean ± SEM, and *p < 0.05, and ***p < 0.001 was determined by unpaired student t-test.

We hypothesized that CHCHD2 up-regulation in HD cells is a compensatory protective response against oxidative stress. To test this hypothesis, we treated human neuronal cells SH-SY5Y and mouse neuronal cells Neuro-2a with the oxidative stress inducer TBHP and hydrogen peroxide (H_2_O_2_). The results revealed that CHCHD2 expression is induced by TBHP in both human and mouse neuronal cells (Fig. 2A, B). Furthermore, CHCHD2 expression was also induced by H_2_O_2_ oxidative stress in SH-SY5Y and Neuro-2a cells (Fig. 2C, D). These results corroborate the notion that CHCHD2 upregulation in HD cells constitutes a protective response against oxidative stress, contributing to the maintenance of cellular homeostasis.

### Increased susceptibility of HD cells to oxidative stress

**Fig. 3:**
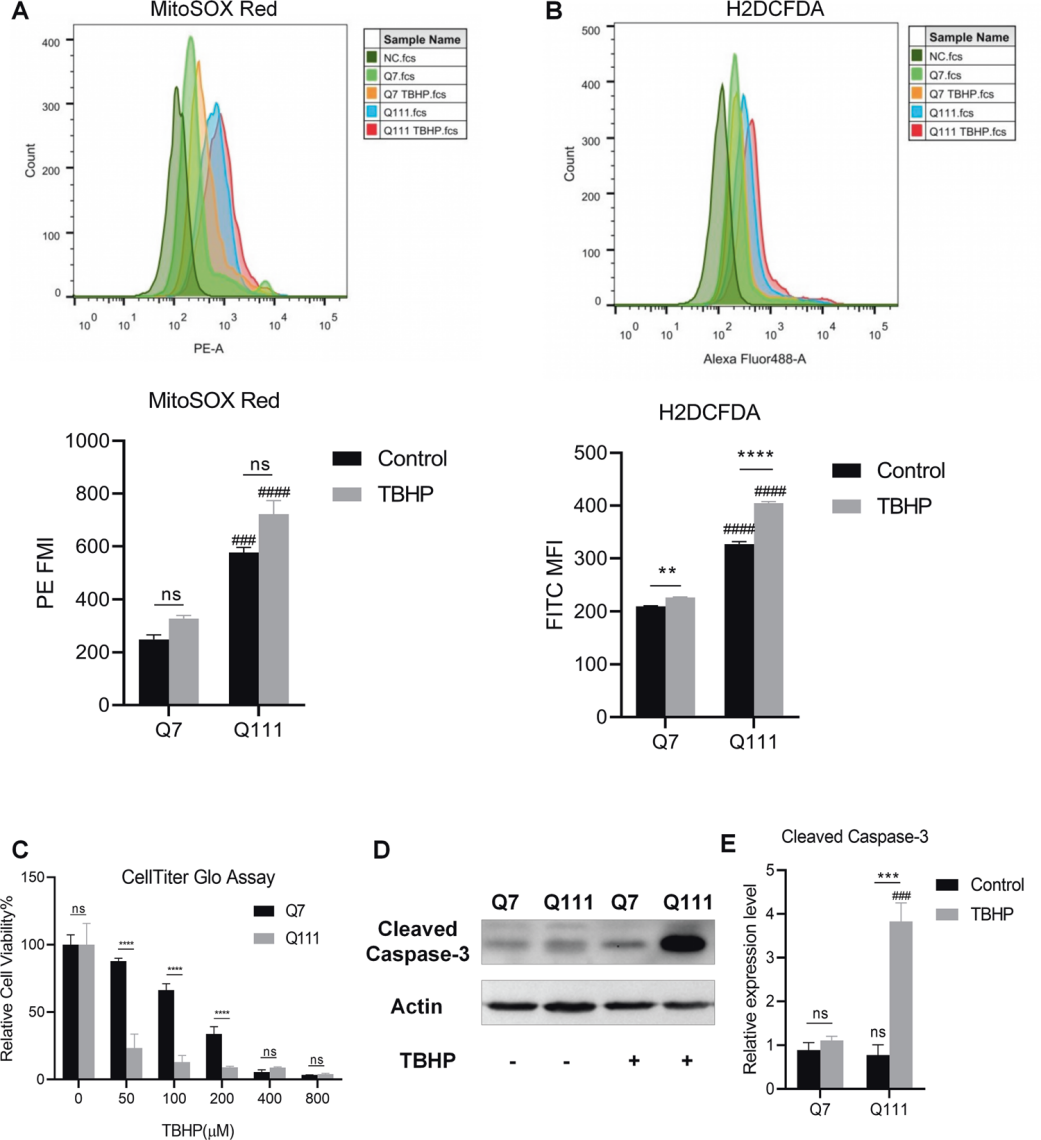
HD cells display increased cellular susceptibility to oxidative stress. **A** Mitochondrial ROS in Q7 and Q111 cells under basal and TBHP treatment conditions are detected by MitoSox Red. **B** Cellular ROS in Q7 and Q111 cells under basal and TBHP treatment conditions are detected by H2DCFDA. *n* = 3 biological replicates for (**A**, **B**). **C** Q7 and Q111 cells were treated with different concentrations (0, 50, 100, 200, 400, and 800 µM) of TBHP for 24 h. Cell viability was determined by CellTiter-Glo assay. *n* = 8 biological replicates. **D**, **E** Cleaved Caspase-3 expression levels in Q7 and Q111 cells after 50 µM THBP treatment for 24 h were determined by immunoblotting. *n* = 3 independent biological replicates; Values shown as mean ± SEM. ns, no significance, **p < 0.01, ***p < 0.001, and ****p < 0.0001 for shown comparisons, and ^###^*p* < 0.001, and ^####^*p* < 0.0001 relative to the corresponding Q7 group was determined by two-way ANOVA analysis followed by a Bonferroni post hoc multiple-comparison test.

We also evaluated the levels of mitochondrial ROS and cellular ROS in Q7 and Q111 cells using the fluorescent dyes MitoSOX Red and H2DCFHDA, respectively. The results indicated higher basal levels of mitochondrial and cellular ROS in Q111 cells compared to Q7 cells (Fig. 3A). In addition, cellular ROS levels were significantly increased after TBHP treatment in both Q7 and Q111 cells (Fig. 3B). Overall, Q111 cells exhibited significant increases in ROS levels and increased susceptibility to oxidative stress compared to Q7 cells. To assess the toxic effect of the oxidative stress inducer TBHP on Q7 and Q111 cells, we employed the CellTiter-Glo assay to measure cell viability. The results showed a dose-dependent decrease in cell viability in both Q7 and Q111 cells. At a concentration of 50 μM TBHP treatment for 24 h, the viabilities of Q7 and Q111 cells decreased by 12.17% and 76.7%, respectively (Fig. 3C). Furthermore, the programmed cell death marker Cleaved Caspase-3 protein was highly expressed in Q111 cells after 50 μM TBHP treatment for 24 h (Fig. 3D, E). These results demonstrate that Q111 cells are more susceptible to oxidative stress compared to Q7 cells.

### Knockdown of CHCHD2 increases ROS levels and reduced cell survival in both WT and HD cells

**Fig. 4:**
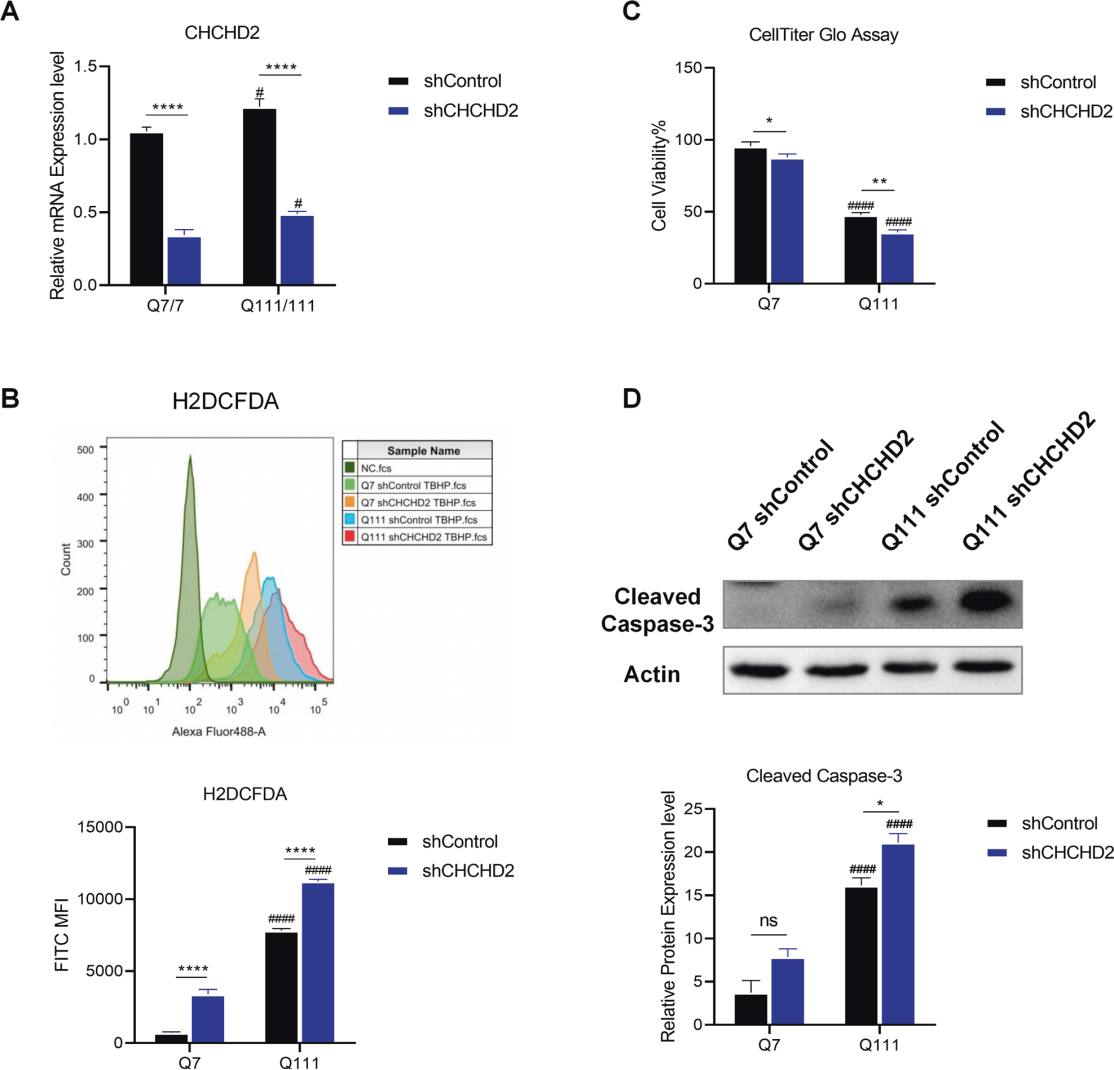
Knockdown of CHCHD2 increases cellular ROS and apoptosis, and deceases cell viability. **A** qRT-PCR results shown that knockdown of endogenous CHCHD2 by lentiviral shRNA reduced the expression of this gene in Q7 and Q111 cells. *n* = 3 independent biological replicates. **B** Cellular ROS was determined by H2DCFDA, and the corresponding mean fluorescence intensity (MFI) with values were shown in the upper histogram and plotted in the graph below. *n* = 3 biological replicates. **C** The cell viability was determined by CellTiter-Glo assay, and the relative cell viability to untreated Q7 shControl group was plotted in the graph. *n* = 7 biological replicates. **D** Cleaved Caspase-3 expression levels in Q7 and Q111 cells knockdown by shControl and shCHCHD2 was determined by immunoblotting. *n* = 3 independent biological replicates; Values shown as mean ± SEM; *p < 0.05, **p < 0.01, and ****p < 0.0001 for shown comparisons, and ^#^*p* < 0.05, ^####^*p* < 0.001, and ^#####^*p* < 0.0001 relative to the corresponding Q7 group was determined by two-way ANOVA analysis followed by a Bonferroni post hoc multiple-comparison test.

We hypothesized that the up-regulation of CHCHD2 is a compensatory protective response to the increased ROS levels in HD cells. To validate this hypothesis, we established stable cell lines using shCHCHD2 lentivirus and shControl lentivirus (Fig. 4A and Supplemental Fig. 2A, B). We then examined cellular ROS levels in these stable cell lines and found that knockdown of CHCHD2 significantly increased cellular ROS levels in both Q7 and Q111 cells, particularly in Q111 cells (Fig. 4B). In addition, knockdown of CHCHD2 significantly decreased cell viability and increased cellular apoptosis after TBHP treatment (Fig. 4C, D). These findings suggest that negative modulation of CHCHD2 through shRNA knockdown enhances oxidative stress-induced apoptosis, highlighting the essential role of CHCHD2 in modulation of ROS levels and cellular protection against oxidative stress in both WT and HD cells.

### Overexpression of CHCHD2 increases HD cell survival under oxidative stress

**Fig. 5:**
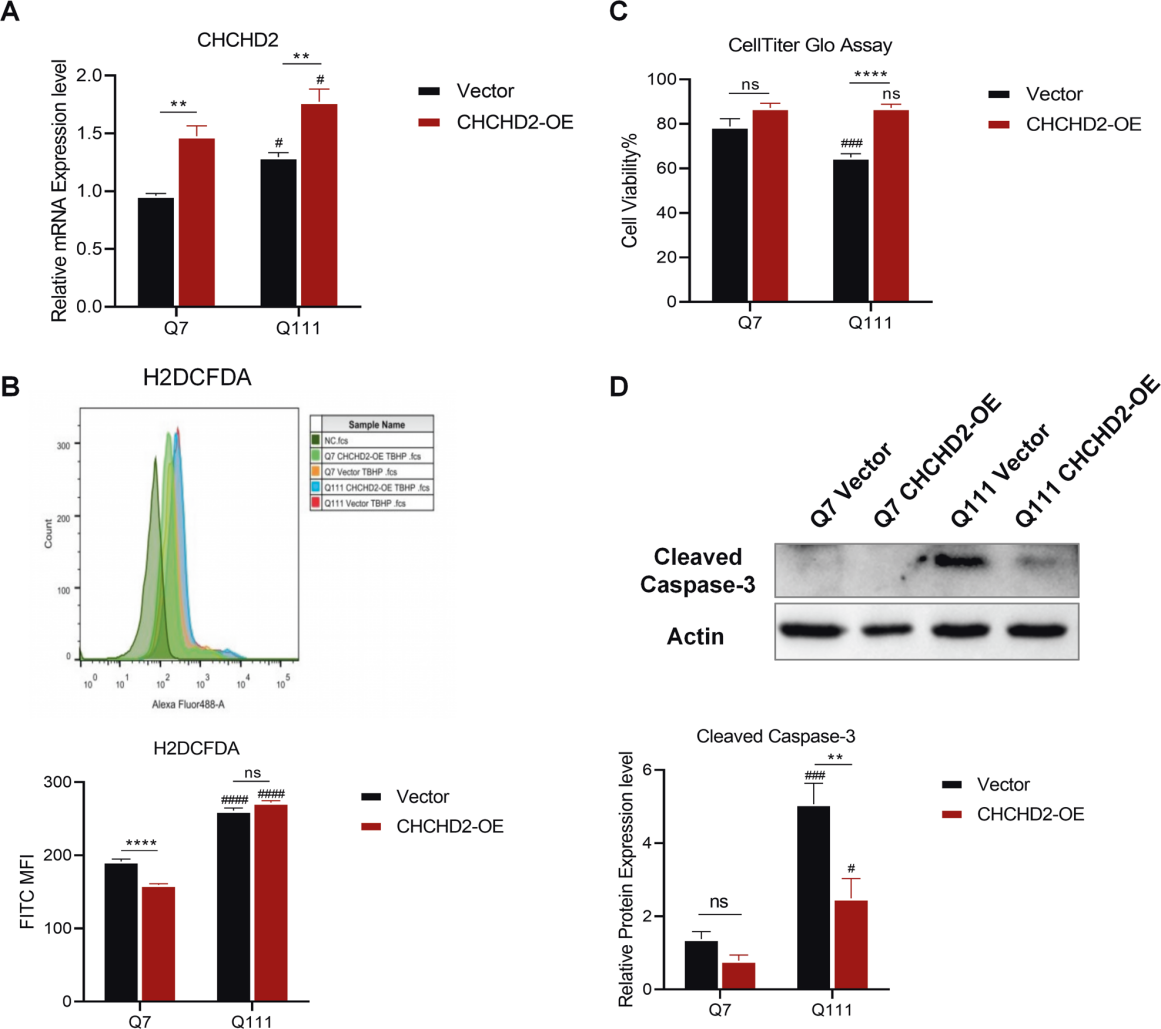
Overexpression of CHCHD2 protects HD cells against TBHP-mediated neuronal damage. **A** Overexpression of Control (Vector) and CHCHD2 (CHCHD2-OE) in Q7 and Q111 cells and quantification by qRT-PCR. *n* = 3 independent biological replicates. **B** Cellular ROS was determined by H2DCFDA, and the corresponding mean fluorescence intensity (MFI) with values was shown in the upper histogram and plotted in the graph below. *n* = 3 biological replicates. **C** The cell viability of Q7 and Q111 cells after 24-h TBHP treatment was determined by CellTiter-Glo assay, and the relative cell viability to untreated Q7 vector group was plotted in the graph. *n* = 7 biological replicates. **D** Cleaved Caspase-3 expression levels in Vector and CHCHD2-OE cells after 24-h TBHP treatment were determined by immunoblotting. *n* = 3 independent biological replicates; Values shown as mean ± SEM; ns, no significance, **p < 0.01, and ****p < 0.001 for shown comparisons, and ^#^*p* < 0.05, ^###^*p* < 0.001, and ^####^*p* < 0.0001 relative to the corresponding Q7 group was determined by two-way ANOVA analysis followed by a Bonferroni post hoc multiple-comparison test.

In order to further confirm the role of CHCHD2 in HD, we used lenti-Vector (Vector) and lenti-CHCHD2 (CHCHD2-OE) overexpression lentiviruses to construct the stable strains of Q7 and Q111. Successful overexpression of CHCHD2 was confirmed by qRT-PCR (Fig. 5A) and Western Blotting (Supplemental Fig. 2C, D). Interestingly, CHCHD2-OE only reduced cellular ROS levels in Q7 cells but not in Q111 cells under oxidative stress (Fig. 5B), indicating a more complex modulation mechanism of ROS levels in HD. Moreover, CHCHD2-OE significantly increased cell survival and decreased cellular apoptosis in Q111 cells under oxidative stress compared to the Vector group (Fig. 5C, D). These results support the protective effect of CHCHD2 in HD cells against high levels of cellular ROS.

### CHCHD2-mediated neuronal protection is associated with Nrf-ARE signaling activation

The Nrf2-ARE pathway is recognized as a crucial endogenous cellular defense mechanism against oxidative stress in neural cells. Considering the observed increase in ROS levels in Q111 cells, we further investigated the activation of the Nrf2-ARE signaling in these cells. We observed elevated Nrf2 expression in Q111 cells compared to Q7 cells (Supplemental Fig. 3A). Moreover, qRT-PCR analysis revealed high mRNA expression levels of Nrf2 downstream target genes (NQO1, HO-1, GCLC, and GCLM) in Q111 cells, with further increases upon exposure to oxidative stress (Supplemental Fig. 3B–E). In addition, the mRNA expression of P62, an autophagy-related gene and modulator of the Nrf2-ARE signaling, was up-regulated in both Q7 and Q111 cells upon TBHP treatment (Supplemental Fig. 3F). These data suggest that the activation of Nrf2-ARE signaling may contribute to the up-regulation of the cellular defense mechanism.

**Fig. 6:**
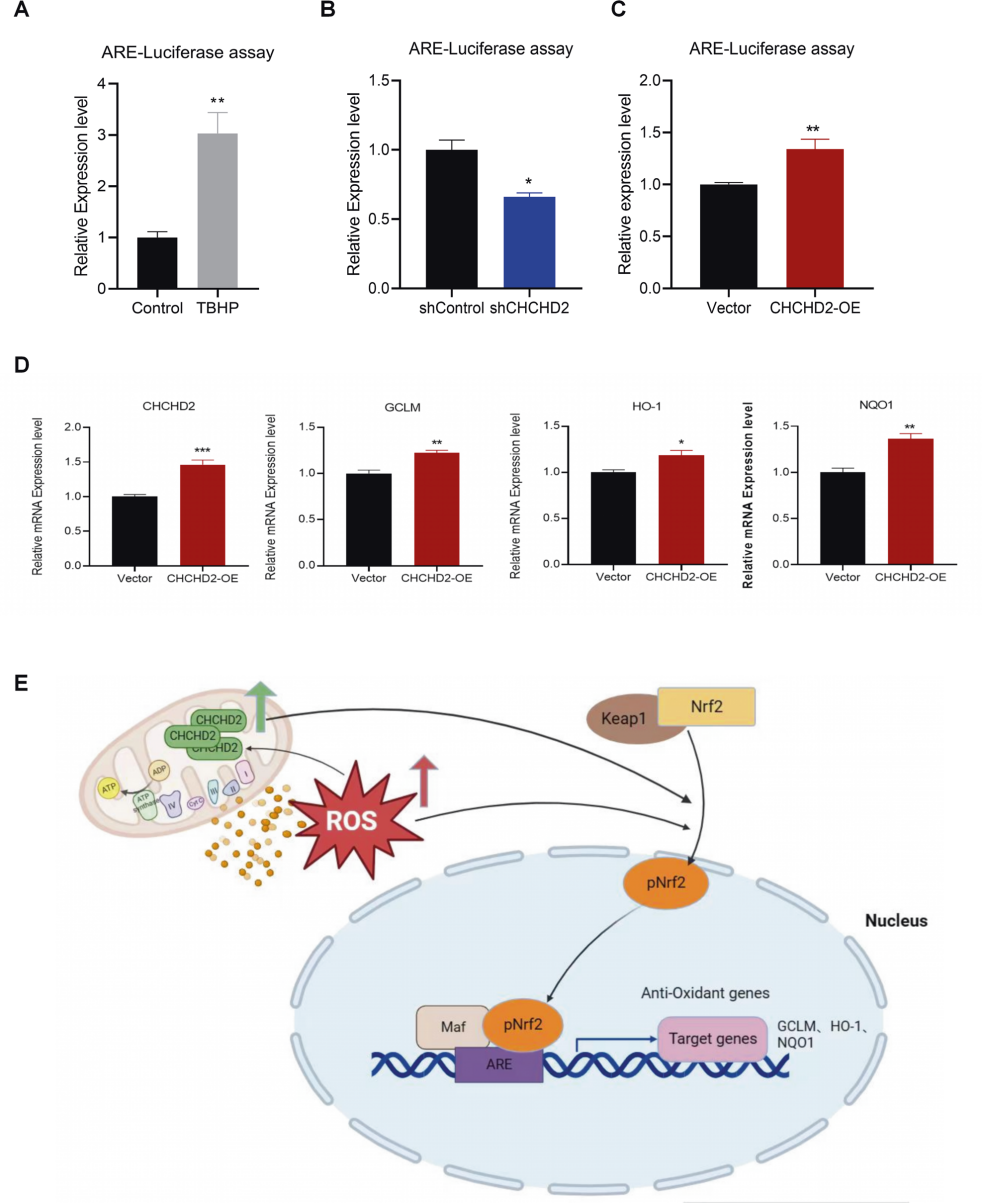
CHCHD2-mediated neuronal protection is associated with the activation of Nrf2-ARE signaling. **A** ARE-luciferase assay was tested by TBHP treatment in SH-SY5Y cells. **B** KD of CHCHD2 resulted in decreased expression of ARE-Luciferase in Q7 cells. **C** OE of CHCHD2 induced up-regulated expression of ARE-Luciferase in Q7 cells. *n* = 3 independent biological replicates for (**A**–**C**). **D** The expression levels of antioxidant target genes (GCLM, HO-1, and NQO1) were increased in Q7 cells with CHCHD2 overexpression. *n* = 4 independent biological replicates. Values shown as mean ± SEM. *p < 0.05, **p < 0.01, and ***p < 0.001 was determined by unpaired student t-test. **E** Schematic of CHCHD2 up-regulation in HD cells mediating a compensatory protective response against oxidative stress.

We propose that CHCHD2 may mediate neuronal protection by activating Nrf2-ARE signaling. To verify this hypothesis, we employed an ARE-Luciferase assay. Firstly, we verified the ARE-luciferase assay by treating SH-SY5Y cells with TBHP, resulting in increased ARE-Luciferase activity (Fig. 6A). Secondly, we observed decreased ARE-Luciferase activity in the shCHCHD2 group compared to the shControl group, indicating that knockdown of CHCHD2 inhibited the activation of Nrf2-ARE signaling (Fig. 6B). Thirdly, overexpression of CHCHD2 up-regulated ARE-Luciferase activity (Fig. 6C). Importantly, overexpression of CHCHD2 in Q7 cells up-regulated Nrf2-ARE downstream target gene expression (Fig. 6D). Overall, these findings suggest that CHCHD2 may mediate neuronal protection by activating Nrf2-ARE signaling (Fig. 6E).

### Validation of CHCHD2’s function in HD using hiPSC-derived neurons

**Fig. 7:**
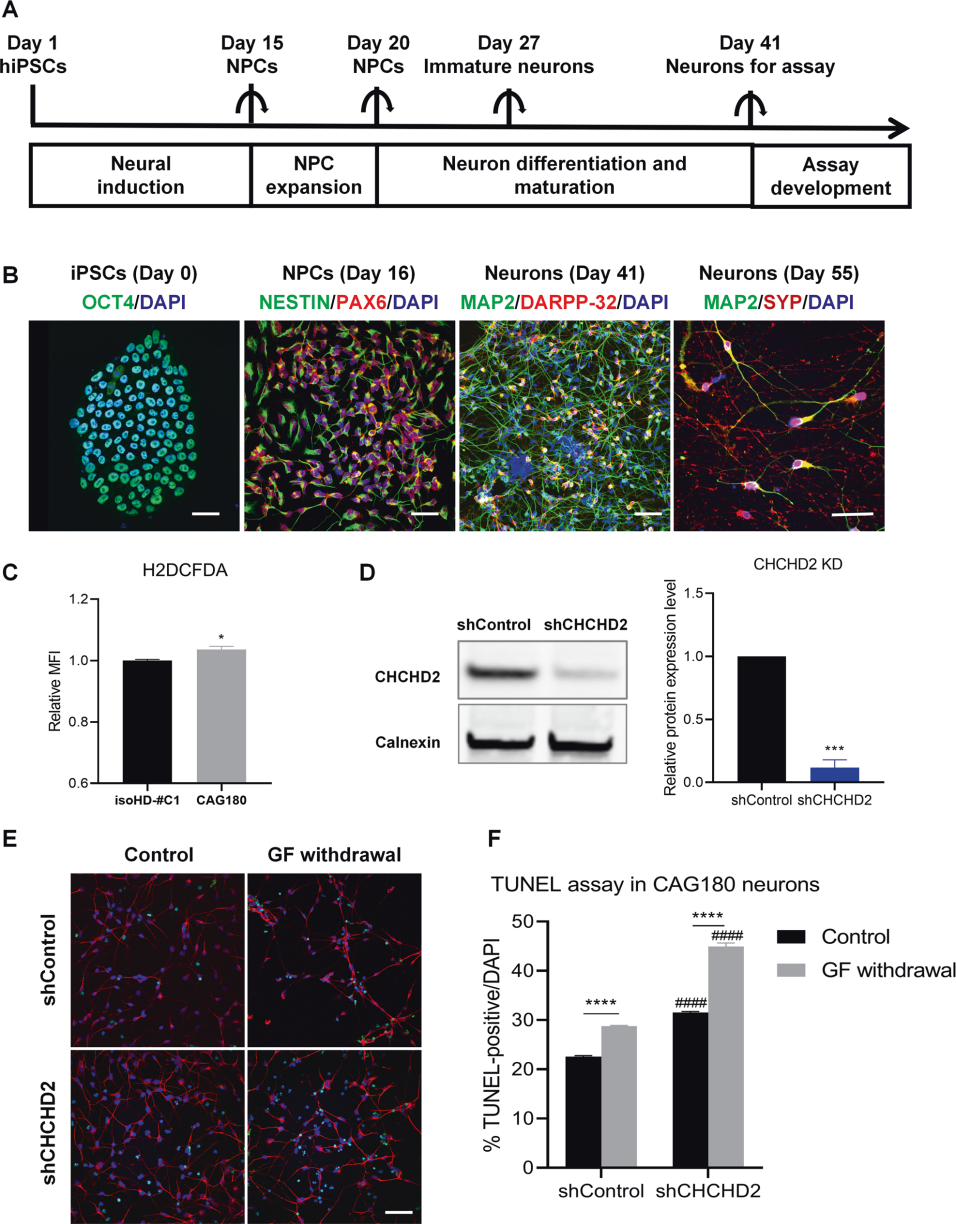
Knockdown of CHCHD2 in HD hiPSC-derived neurons. **A** Scheme of forebrain neuron differentiation protocol. **B** Representative images of hiPSC marker OCT4 (green), NPC marker NESTIN (green) and PAX6 (red), post-mitotic neuron marker MAP2 (green), striatal maker DARPP-32 (red), synaptic marker Synaptophysin (SYP, red) and DNA marker DAPI (blue). Scale bar, 50 µm. **C** Cellular ROS in CAG180 iPSC-derived neurons (HD) and its isogenic control line isoHD-#C1 iPSC-derived neurons (Control) was detected by H2DCFDA. *n* = 4 independent biological replicates; *p < 0.05, was determined by unpaired student t-test. **D** Knockdown of CHCHD2 in CAG180 hiPSC-derived neurons (Day 41) by lentiviral shControl and shCHCHD2. *n* = 3 independent biological replicates; *p < 0.05, and ***p < 0.001 was determined by unpaired student t-test for (**C**) and (**D**). **E** Representative images of GF (growth factor) withdrawal assay. TUNEL was shown in green, MAP2 was shown in red, and DNA marker DAPI was in blue. Scale bar, 50 µm. **F** The percentage of TUNEL ( + )/DAPI was quantified. *n* = 3 independent biological replicates. Values shown as mean ± SEM. ****p < 0.0001 for shown comparisons, and ^#####^*p* < 0.0001 relative to the corresponding shControl group was determined by two-way ANOVA analysis followed by a Bonferroni post hoc multiple-comparison test.

Considering the crucial roles of CHCHD2 in modulating cellular ROS and mediating anti-apoptosis function in murine striatal cell lines, we further validated whether CHCHD2 plays similar roles in HD iPSC-derived neurons. Figure 7A shows the differentiation of CAG180 (HD) and its isogenic Control (isoHD-#C1) iPSCs into forebrain neurons using an established protocol. Immunofluorescence characterization of the cells during different stages of neural induction and differentiation is shown in Fig. 7B. Generally, OCT4(+) hiPSCs were differentiated into NESTIN( + )/PAX6(+) NPCs in neural induction medium. These hiPSC-derived NPCs were further differentiated into MAP2(+) neurons, some of which expressed the striatal marker DARPP32. By Day 55 of neuronal differentiation, hiPSC-derived neurons expressed the synaptic marker SYP, indicating their maturation into functional neurons.

Importantly, our results demonstrated higher cellular ROS levels in HD neurons compared to Control neurons (Fig. 7C). Furthermore, we knocked down CHCHD2 in CAG180 iPSC-derived neurons (Fig. 7D) and conducted a TUNEL assay. Our findings showed that CHCHD2 knockdown affected neuronal survival in post-mitotic CAG180 iPSC-derived neurons, indicating a protective role for CHCHD2 in the context of mutant HTT in HD neurons (Fig. 7E, F). Together, our finding in HD iPSCs model also supported the previously proposed hypothesis of CHCHD2 up-regulation in HD mediating a compensatory protective response against oxidative stress.

## Discussion

Oxidative stress-mediated neuronal cell death has been implicated in various neurodegenerative disorders including PD, AD, amyotrophic lateral sclerosis (ALS), and HD [26]. Oxidative stress plays a crucial role in the pathogenesis of HD, making it a key target for HD treatment [27]. In this study, we observed high expression of CHCHD2 in HD striatal cells. Overexpression of CHCHD2 reduced intracellular ROS levels, enhanced cell survival under oxidative stress, and exerted a neuroprotective effect associated with activation of the Nrf2-ARE pathway. These findings highlight the anti-oxidative stress function of CHCHD2 and provide new insights for HD treatment.

The Nrf2/ARE pathway is a critical anti-oxidative stress pathway in neurodegenerative diseases. Activation of Nrf2 has been shown to protect cortical and striatal neurons, reduce motor deficits, and extend lifespan in animal models of HD [28]. Selective activation of Nrf2 signaling effectively inhibits the release of the pro-inflammatory factor interleukin-6 in primary microglia and astrocytes from HD and wild-type mice [29]. Moreover, in primary monocytes from healthy individuals and HD patients, Nrf2 suppresses the expression of pro-inflammatory cytokines, including interleukin-1, interleukin-6, interleukin-8, and tumor necrosis factor alpha [29]. These findings collectively support the protective potential of the Nrf2 signaling pathway in key cell types associated with HD pathology, making Nrf2 a potential molecular target for HD treatment. A recent study demonstrated increased expression of Nrf2 and its downstream targets NQO1, HO-1, and GCLM mRNA in an HD model using PC12 cells [30]. Consistent with this, our experiments also confirmed elevated mRNA expression levels of Nrf2, NQO1, HO-1, GCLC, and GCLM in HD mouse cell lines, where Q111 cells exhibited significantly higher expression compared to Q7 (Supplemental Fig. 3), indicating activation of the Nrf2-ARE anti-oxidative defense system in HD cells.

CHCHD2 has recently been identified as a regulator of mitochondrial metabolism [10, 31]. Aras et al. demonstrated that CHCHD2 plays a role in the mitochondrial intermembrane space by activating cytochrome c oxidase (COX) and in the nucleus by promoting the transcription of specific genes, including COX4I2 and itself, in response to hypoxia [9]. Up-regulation of CHCHD2 has been observed in HD hESCs, hiPSCs, NSCs and human HD peripheral blood cells [18–20, 22]. In our study, KD of CHCHD2 in HD neurons reduced neuronal survival under both normal and growth factor withdrawal conditions. This suggests that the upregulation of CHCHD2 in HD may serve as a compensatory response to mutant HTT, and CHCHD2 could potentially serve as a novel molecular marker of HD pathology. Our findings in this study provide experimental evidence linking CHCHD2 to neuronal death in HD, and point to the potential of CHCHD2 as a prospective drug development target for HD.

Considering the potential anti-oxidative effect of CHCHD2 upregulation, it is essential to further clarify the specific transcription factors and pathways regulating CHCHD2 expression and function. Ruan et al. showed that the mitochondrial proteases OMA1 and Yme1L, which have been shown to interact with CHCHD2, are responsible for its degradation under certain conditions [32]. In addition, Wei et al. experimentally verified the interaction between CHCHD2 and p32/C1QBP, a multifunctional mitochondrial chaperone implicated in oxidative stress responses [33, 34], highlighting its close association with the generation of ROS and the oxidative stress process [35]. Furthermore, Liu et al. revealed that CHCHD2 plays a role in regulating the levels of Opa1, a key determinant of mitochondrial dynamics and energetics, by competing with Yme1L for p32/C1QBP binding [36]. These observations suggest complex post-translational processes regulating CHCHD2 expression and function. However, less is known about the transcriptional mechanisms that influence CHCHD2 expression. Future investigations could explore the involvement of well-known oxidative stress-responsive transcription factors, such as *Nrf2* and *HIF-1α*, and signaling pathways, like MAPK and PI3K/AKT in this process. Such studies could help clarify the mechanisms underlying changes in CHCHD2 expression in HD and would contribute to a more comprehensive understanding of the role CHCHD2 in normal physiology and disease.

The establishment of animal models has been instrumental in studying the pathogenesis of HD [37]. However, there are inherent limitations due to differences between humans and animals in terms of anatomy, physiological functions, and the timing and progression of the disease. The emergence of hiPSCs has partially overcome the limitations of animal models [38]. hiPSCs can be derived from the adult cells of HD patients, carrying endogenously expressed full-length mutated HTT proteins and sharing the exact same genetic background as the patients [39]. In the HD hiPSC model, the most stringent control group consists of isogenic controls created using CRISPR technology. The use of isogenic controls ensures that the HD and control groups are derived from the same parental cell line and are otherwise identical except for the mutated HTT gene. In our study, we used such isogenic controls for the HD hiPSC lines [20]. In this study, we observed increased ROS levels and cellular susceptibility to growth factor withdrawal in HD hiPSC-derived neuronal cells. Furthermore, overexpression of CHCHD2 protected HD hiPSC-derived neurons against growth factor deprivation.

## Conclusions

In this study, we have demonstrated that both oxidative stress and CHCHD2 expression are increased in HD models. Furthermore, we have observed that CHCHD2 expression is highly induced by oxidative stress in both human and mouse neuronal cells. Through our experiments, we have confirmed that overexpression of CHCHD2 provides protection to neuronal cells against oxidative stress-induced damage. This protective effect of CHCHD2 is associated with the activation of the Nrf2-ARE signaling pathway, indicating that the up-regulation of CHCHD2 in HD serves as a compensatory response against oxidative stress. Our results suggest that positive modulation of CHCHD2-mediated protective mechanism against oxidative damage in neuronal cells may provide novel neuroprotective strategies for treatment of HD and other neurodegenerative diseases.

### Supplementary information

Reporting Checklist

Supplemtary file for Original western blotting images

Supplemental Figures and Tables

## Data Availability

All data generated or analyzed during this study are available from the corresponding author on reasonable request.
