# Supplementary material for: CHCHD2 up-regulation in Huntington disease mediates a compensatory protective response against oxidative stress
Source: Cell Death Dis. 2024 Feb 10;15(2):126. doi: 10.1038/s41419-024-06523-x (PMC10858906; doi:10.1038/s41419-024-06523-x)
Supplement: Supplementary file 3 — Supplemental Figures and Tables [file 41419_2024_6523_MOESM3_ESM.docx]

**Supplementary Information**

**Supplemental Figures and Legends**

| 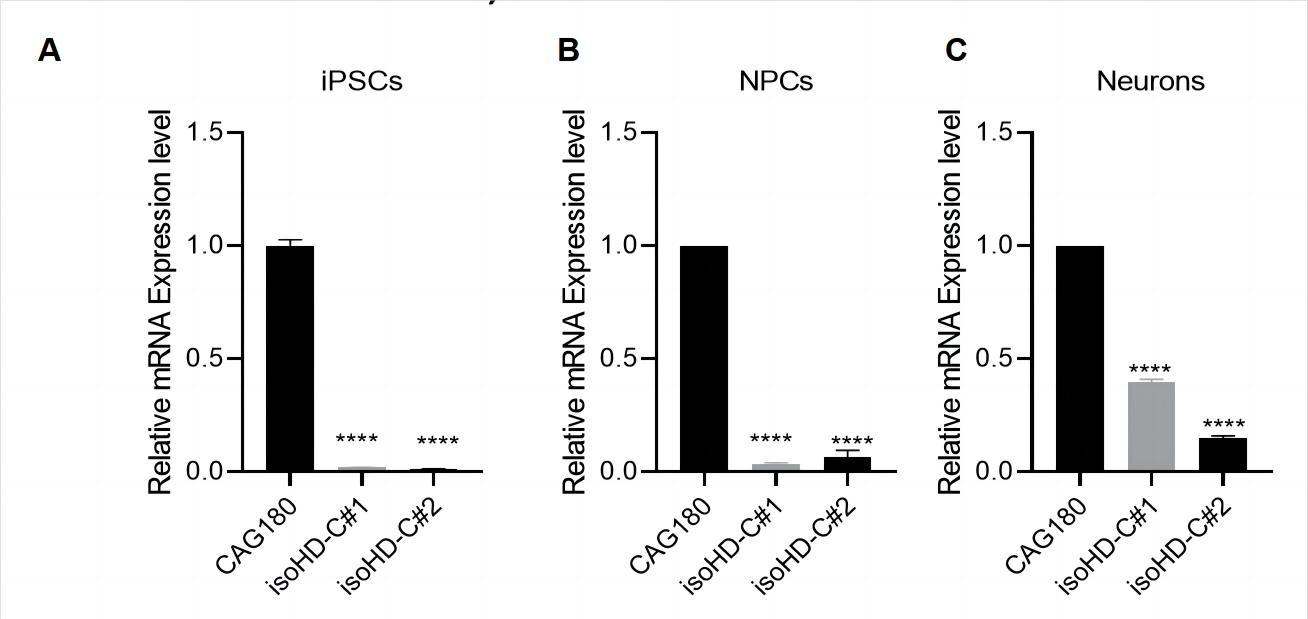 |
| --- |
| **Supplemental Fig. 1 CHCHD2 is highly expressed in HD iPSCs and their-derived neuronal cells.**  **(A-C)** mRNA expression levels of CHCHD2 at the different stages of neural induction and neuronal differentiation were measured by qRT-PCR. iPSCs, Day 0; NPCs, Day 15; Neurons, Day 34. n = 3 biological replicates; Values shown as mean ± SEM; **** p < 0.0001 relative to CAG180 group was determined by unpaired student t-test. |

| 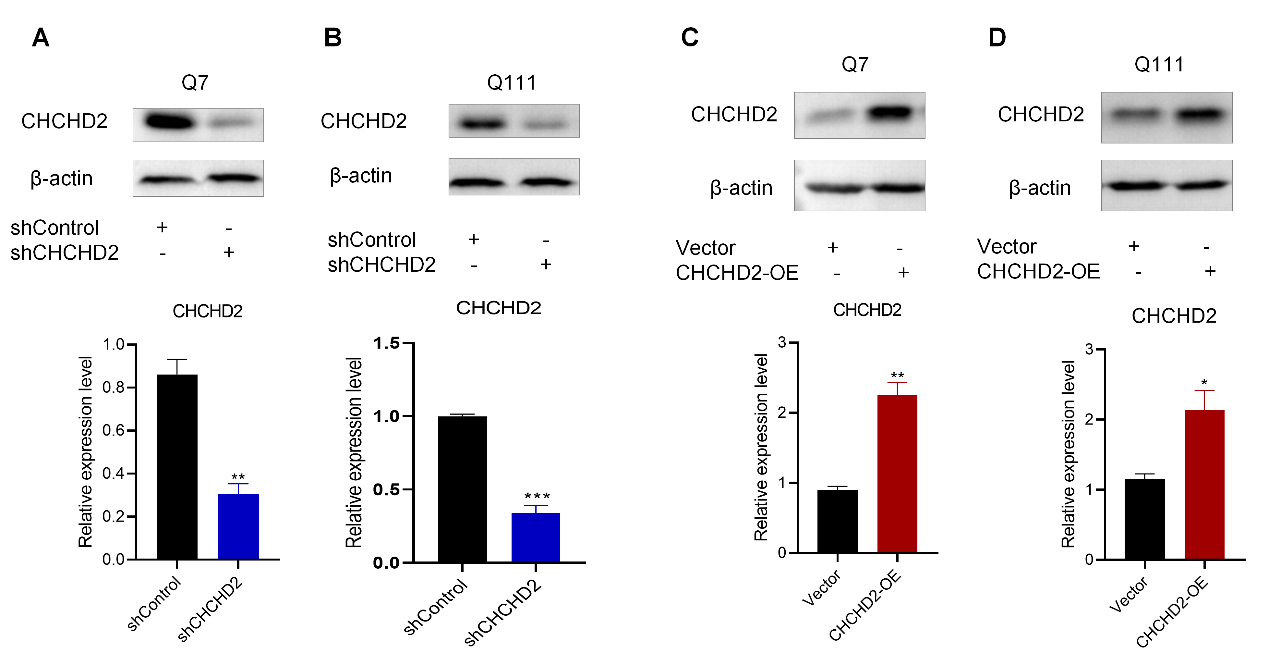 |
| --- |
| **Supplemental Fig. 2 Construction of stable Q7 and Q111 cells with CHCHD2 knockdown and overexpression**  **(A)** Knockdown of CHCHD2 in Q7 cells, and CHCHD2 protein expression was measured by immunoblotting. **(B)** Knockdown of CHCHD2 in Q111 cells, and CHCHD2 protein expression was measured by immunoblotting. **(C)** Overexpression of CHCHD2 in Q7 cells, and CHCHD2 protein expression was measured by immunoblotting. **(D)** Overexpression of CHCHD2 in Q111 cells, and CHCHD2 protein expression was measured by immunoblotting. n = 3 biological replicates; Values shown as mean ± SEM; * p < 0.05, ** p < 0.01, and *** p < 0.001 for shown comparisons was determined by unpaired student t-test. |

| 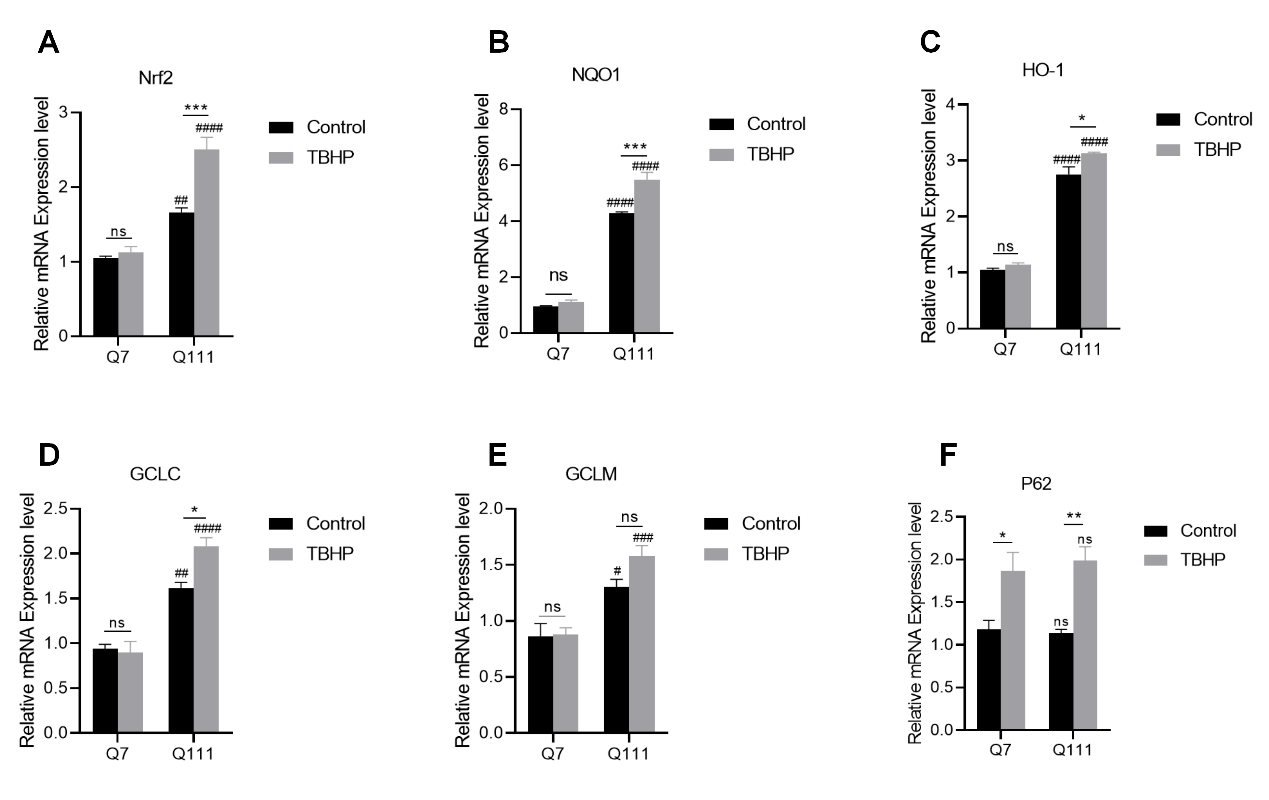 |
| --- |
| **Supplemental Fig. 3 The Nrf2-ARE anti-oxidative stress signaling is activated in HD cells.**  (**A-F**) The mRNA expression levels of Nrf2 (**A**), Nrf2-ARE signaling target genes (NQO1, HO1, GCLC, and GCLM) (**B-E**), and autophagy related gene P62 (**F**) were measured by qRT-PCR. n = 3 independent biological replicates; Values shown as mean ± SEM; ns, no significance, * p < 0.05, ** p < 0.01, and *** p < 0.001 for shown comparisons, and # p < 0.05, ## p < 0.01, ### p < 0.001, and #### p < 0.0001 relative to the corresponding Q7 group was determined by two-way ANOVA analysis followed by a Bonferroni post hoc multiple-comparison test. |

**Supplemental Table 1. Sequences of primers for qRT-PCR analysis**

| Name | Species | Forward (5ʹ→3ʹ) | Reverse (5ʹ→3ʹ) |
| --- | --- | --- | --- |
| *ACTIN* | *human* | *GTCTTCCCCTCCATCGTG* | GATGGGGTACTTCAGGGTGA |
| *CHCHD2* | *human* | *GCTTCAGTGGAGGAAGTAATG* | *TGATGTCACCCTGGTTCT* |
| *ACTIN* | *mouse* | *GGCTGTATTCCCCTCCATCG* | CCAGTTGGTAACAATGCCATGT |
| *CHCHD2* | *mouse* | TCCTCTCTCCTTGGTAGAAAT | CATCCTAACTGCTCGAAGTC |
| *HTT* | *mouse* | GTGCTCCTCGAAGTTTGCGT | GATTCCTCCGGTCTTTTGCTT |
| Nrf2 | *mouse* | CAACTCGGCGAAGAAAGAAACA | AGGATACTGGGGATTCGTCTG |
| *NQO1* | *mouse* | AGGATGGGAGGTACTCGAATC | AGGCGTCCTTCCTTATATGCTA |
| *HO-1* | *mouse* | TCGTGCTCGAATGAACACTCTGGA | TGTGTTCCTCTGTCAGCATCACCT |
| *GCLC* | *mouse* | GGGGTGACGAGGTGGAGTA | GTTGGGGTTTGTCCTCTCCC |
| *GCLM* | *mouse* | AGGAGCTTCGGGACTGTATCC | GGGACATGGTGCATTCCAAAA |
| *P62* | *mouse* | TGAAACATGGACACTTTGGCTGGC | ACATTGGGATCTTCTGGTGGAGCA |
