# Supplementary material for: CHCHD2 up-regulation in Huntington disease mediates a compensatory protective response against oxidative stress
Source: Cell Death Dis. 2024 Feb 10;15(2):126. doi: 10.1038/s41419-024-06523-x (PMC10858906; doi:10.1038/s41419-024-06523-x)
Supplement: Supplementary file 2 — Supplemtary file for Original western blotting images [file 41419_2024_6523_MOESM2_ESM.docx]

Original western blotting images

Supplementary Figure 1. Original western blotting images for Figure 1C

**Q7/111**

**Q111**

**Q7**

**Q111**

**Q7**

**Q7/111**

**Q7/111**

**Q111**

**CHCHD2 17KD**

**Actin 42KD**

**10KD-**

**15KD-**

**Repeat 1**

**Repeat 2**

**Repeat 3**

**Q7**

Supplementary Figure 2. Original western blotting images for Figure 1E

**Q7**

**Q111**

**HTT ~350KD**

**Calnexin 90KD**

**Q7**

**Q111**

**Q7**

**Q111**

**75KD-**

**100KD-**

**245KD-**

**Repeat 1**

**Repeat 2**

**Repeat 3**

Supplementary Figure 3. Original western blotting images for Figure 2A

**Actin 42KD**

**CHCHD2 17KD**

**15KD-**

**Control**

**TBHP**

**Control**

**TBHP**

**Control**

**TBHP**

**35KD-**

**40KD-**

**50KD-**

**Repeat 1**

**Repeat 2**

**Repeat 3**

**Control**

**TBHP**

**Control**

**TBHP**

**Control**

**TBHP**

**Repeat 4**

**Repeat 5**

**Repeat 6**

Supplementary Figure 4. Original western blotting images for Figure 2B

**Actin 42KD**

**35KD-**

**40KD-**

**50KD-**

**CHCHD2 17KD**

**15KD-**

**Control**

**TBHP**

**Control**

**TBHP**

**Control**

**TBHP**

**Repeat 1**

**Repeat 2**

**Repeat 3**

**Control**

**TBHP**

**Control**

**TBHP**

**Control**

**TBHP**

**Repeat 4**

**Repeat 5**

**Repeat 6**

Supplementary Figure 5. Original western blotting images for Figure 2C

**Repeat 3**

**Repeat 1**

**Repeat 2**

**NA**

**NA**

**Repeat 4**

**CHCHD2 17KD**

**H2O2**

**Control**

**H2O2**

**Control**

**Control**

**H2O2**

**Control**

**H2O2**

**8KD-**

**17KD-**

**25KD-**

**Actin 42KD**

**43KD-**

**33KD-**

**55KD-**

Supplementary Figure 6. Original western blotting images for Figure 2D

**Repeat 3**

**Repeat 2**

**Repeat 1**

**Control**

**Repeat 4**

**CHCHD2 17KD**

**H2O2**

**H2O2**

**H2O2**

**H2O2**

**Control**

**Control**

**Control**

**8KD-**

**17KD-**

**25KD-**

**Actin 42KD**

**43KD-**

**33KD-**

**55KD-**

Supplementary Figure 7. Original western blotting images for Figure 3D

**Cleaved**

**Caspase-3 17KD**

**Actin 42KD**

**35KD-**

**40KD-**

**50KD-**

**15KD-**

**20KD-**

**25KD-**

**Q7**

**Q111**

**Q7+TBHP**

**Q111+TBHP**

**Q7**

**Q111**

**Q7+TBHP**

**Q111+TBHP**

**Q7**

**Q111**

**Q7+TBHP**

**Q111+TBHP**

**Repeat 1**

**Repeat 2**

**Repeat 3**

Supplementary Figure 8. Original western blotting images for Figure 4D

**Q7 shCHCHD2+TBHP**

**Q7 shControl+TBHP**

**Q111 shControl+TBHP**

**Q111 shCHCHD2+TBHP**

**Q7 shCHCHD2+TBHP**

**Q7 shControl+TBHP**

**Q111 shControl+TBHP**

**Q111 shCHCHD2+TBHP**

**Q7 shCHCHD2+TBHP**

**Q7 shControl+TBHP**

**Q111 shControl+TBHP**

**Q111 shCHCHD2+TBHP**

**Actin 42KD**

**35KD-**

**40KD-**

**50KD-**

**60KD-**

**Cleaved**

**Caspase-3 17KD**

**15KD-**

**20KD-**

**25KD-**

**Repeat 1**

**Repeat 2**

**Repeat 3**

Supplementary Figure 9. Original western blotting images for Figure 5D

**Actin 42KD**

**35KD-**

**40KD-**

**50KD-**

**60KD-**

**Q7 Vector+TBHP**

**Q7 CHCHD2-OE+TBHP**

**Q111 Vector+TBHP**

**Q111 CHCHD2-OE+TBHP**

**Q7 Vector+TBHP**

**Q7 CHCHD2-OE+TBHP**

**Q111 Vector+TBHP**

**Q111 CHCHD2-OE+TBHP**

**Q7 Vector+TBHP**

**Q7 CHCHD2-OE+TBHP**

**Q111 Vector+TBHP**

**Q111 CHCHD2-OE+TBHP**

**Repeat 1**

**Repeat 2**

**Repeat 3**

**Cleaved**

**Caspase-3 17KD**

**15KD-**

**20KD-**

**25KD-**

Supplementary Figure 10. Original western blotting images for Figure 7D

250 KD-

100 KD-

50KD-

37KD-

25 KD-

15 KD-

**shControl**

**shCHCHD2-1**

**shCHCHD2-2**

**shControl**

**shCHCHD2-1**

**shCHCHD2-2**

**shControl**

**shCHCHD2-1**

**shCHCHD2-2**

**Calnexin 90KD**

**CHCHD2 17KD**

**Repeat 1**

**Repeat 2**

**Repeat 3**

Supplementary Figure 11. Original western blotting images for Supplemental Figure 2A-B

**Repeat 1**

**Repeat 2**

**Repeat 3**

**Q111 shControl**

**Q111 shControl**

**Q111 shCHCHD2**

**Q111 shControl**

**Q111 shCHCHD2**

**Q111 shCHCHD2**

**Repeat 3**

**Repeat 2**

**Repeat 1**

**Q7 shControl**

**Q7 shCHCHD2**

**Q7 shCHCHD2**

**Q7 shControl**

**Q7 shCHCHD2**

**Q7 shControl**

**60KD-**

**50KD-**

**Actin 42KD**

**35KD-**

**40KD-**

**CHCHD2 17KD**

**10KD-**

**15KD-**

**20KD-**

Supplementary Figure 12. Original western blotting images for Supplemental Figure 2C-D

**Repeat 3**

**Q7 CHCHD2-OE**

**Repeat 2**

**Repeat 1**

**Repeat 1**

**Q111 Vector**

**Repeat 3**

**Repeat 2**

**Q111 Vector**

**Q111 CHCHD2-OE**

**Q111 CHCHD2-OE**

**Q111 Vector**

**Q111 CHCHD2-OE**

**Q7 CHCHD2-OE**

**Q7 Vector**

**Q7 CHCHD2-OE**

**Q7 Vector**

**Q7 Vector**

**Actin 42KD**

**35KD-**

**40KD-**

**50KD-**

**60KD-**

**CHCHD2 17KD**

**10KD-**

**15KD-**

**20KD-**
